# Replacement of the Human Topoisomerase Linker Domain with the Plasmodial Counterpart Renders the Enzyme Camptothecin Resistant

**DOI:** 10.1371/journal.pone.0068404

**Published:** 2013-07-02

**Authors:** Barbara Arnò, Ilda D’Annessa, Cinzia Tesauro, Laura Zuccaro, Alessio Ottaviani, Birgitta Knudsen, Paola Fiorani, Alessandro Desideri

**Affiliations:** 1 Department of Biology and Interuniversity Consortium, National Institute Biostructure and Biosystem (INBB), University of Rome Tor Vergata, Rome, Italy; 2 Department of Molecular Biology and Genetics, Aarhus University, Aarhus, Denmark; 3 Institute of Translational Pharmacology, National Research Council, CNR, Rome, Italy; Institut Pasteur, France

## Abstract

A human/plasmodial hybrid enzyme, generated by swapping the human topoisomerase IB linker domain with the corresponding domain of the *Plasmodium falciparum* enzyme, has been produced and characterized. The hybrid enzyme displays a relaxation activity comparable to the human enzyme, but it is characterized by a much faster religation rate. The hybrid enzyme is also camptothecin resistant. A 3D structure of the hybrid enzyme has been built and its structural-dynamical properties have been analyzed by molecular dynamics simulation. The analysis indicates that the swapped plasmodial linker samples a conformational space much larger than the corresponding domain in the human enzyme. The large linker conformational variability is then linked to important functional properties such as an increased religation rate and a low drug reactivity, demonstrating that the linker domain has a crucial role in the modulation of the topoisomerase IB activity.

## Introduction

Members of the eukaryotic DNA topoisomerases I family are highly conserved enzymes which displays a critical role in fundamental biological processes such as replication, recombination, and transcription [1-4]. Human topoisomerase IB (hTop1) is a 91 KDa monomer, composed by four structural domains: the N-terminal (1-214), the core (215-635), the linker (636-712) and the C-terminal domain (713-765) [5,6]. The enzyme catalyzes the relaxation of supercoiled DNA by first “clamping” a double-stranded DNA and then breaking a single strand forming a transient phosphotyrosine bond between the active site Tyr723 and the 3’ end of the cleaved DNA strand. The covalently bound enzyme holds one end of the DNA duplex, allowing the 5’ end downstream of the cleaved site to rotate around the intact strand. The relaxation occurs through a "controlled rotation" mechanism regulated by ionic interactions between the DNA and the positive charged linker domain, which slows down the free rotation of the DNA, guiding the filament in a position suitable for the next step [7]. The supercoils are removed in this process and the DNA linking number is changed [8]. A second nucleophilic attack, driven by the free 5’-OH, restores an intact double-stranded DNA. hTop1 has a relevant clinical interest since it is the cellular target of several natural compounds [9]. The most important one being the chemotherapeutic agent camptothecin (CPT) and its derivatives such as Topotecan (TPT) and SN38 [10], which all convert the enzyme into a cellular poison by reversibly stabilizing the Top1-DNA covalent complex. CPT intercalates the DNA duplex, moving the 5’-OH end of the DNA away and slowing down the religation step [11].

The linker domain has been shown to be crucial in controlling the enzyme function. Hence, although Top1 retains its *in vitro* activities after removal of the linker domain, the enzyme passes from a processive to a distributive DNA relaxation upon linker deletion [12], suggesting the loss of inter-domain communication, as also indicated by an all atom molecular dynamics (MD) simulation of the enzyme in the presence or absence of the linker [13]. The enzyme depleted of the linker has been also shown to have a faster religation kinetics and to be CPT resistant, highlighting for the first time the importance of the linker in controlling the religation and modulating the CPT sensitivity [12]. The linker has been shown to be in direct contact with the DNA since it is more resistant to proteolysis when the enzyme is non-covalently bound to duplex DNA than in the absence of DNA [5]. Moreover, it is one of the most flexible protein regions, as evidenced by multiple non-isomorphous crystal structures [14] and MD simulation [13,15].

The important role of the linker domain in modulating the enzyme activity and the reactivity towards CPT has been confirmed by the investigation of a drug resistant mutant having a single residue located in the linker namely Alanine 653 mutated in Proline [16]. This mutant has been shown to sample a large conformational space correlated to an increased religation rate that does not permit CPT to stabilize the covalent hTop1-DNA-cleavage complex. The correlation between linker flexibility and CPT reactivity seems to be a general rule since sampling of a decreased conformational space has been reported for the CPT hypersensitive mutant Asp677Gly-Val703Ile [17,18]. In addition binding of the drug reduces the sampled conformational space, independently if it belongs to the CPT or to the structurally unrelated indenoisoquinoline drug family, as confirmed by molecular dynamics simulation of the hTop1-DNA-TPT or of the hTop1-DNA-IQN ternary complex [19,20]. In line the linker domain electron density map is observed in the X-ray diffraction study of the ternary but not of the binary complex [21].

The linker is also involved in modulating the protein inter-domain communication since mutation of Thr718, close to the catalytic Tyr723 residue, induces altered linker flexibility [22]. On the other hand, mutation of the conserved Lys681 residue, located over the linker domain, perturbs the linker dynamics and reduces the enzyme religation rate [23]. Mutation of Thr729 to Lys also abolishes the intra-protein communications between the C-terminal and the linker domain, altering the interactions between helix 17 in the core domain and helix 19 in the linker domain [24,25]. Moreover, mutation of the *S. cerevisiae* enzyme residue Gly721, corresponding to the human Gly717, affects CPT sensitivity and activity of the yeast protein [26]. Inter-domain communications between the linker and other protein domains have been confirmed by means of a long time scale coarse grained molecular dynamics simulation [27], while non-equilibrium molecular dynamics simulations have identified distinct mechanisms for positive and supercoiled DNA relaxation, where the linker domain plays an essential role [28]. Evidences for a long range communication between the linker and the active site regions come from the double mutant Ala653Pro-Thr718Ala, involving Ala653 located on the linker domain and Thr718, located in proximity of the active site. The two mutations abolish the lethal phenotype of the single mutant Thr718Ala, demonstrating how a mutation on the linker domain can influence the active site region [29]. Finally, it has been reported that the linker must retain a minimum length in order to have a fully functional enzyme [30].

Despite all these evidences for a crucial role of the linker domain, either in modulating the enzyme activity or the drug reactivity, a multiple sequence alignment of topoisomerase IB from different species indicates that the linker length as well as its amino acids composition is quite variable [23]. This finding may open the way in exploiting the structural-dynamical properties of linkers from topoisomerase IB from different species to test their role in drug reactivity with the final aim to develop drugs selectively targeting topoisomerase IB from different species [31]. With this aim in mind we have characterized the linker domain of Top1 from the malaria causing pathogenic organism, *Plasmodium falciparum*.

In the present work we have produced and characterized an hybrid enzyme, from here after called hTop1(pf-Linker), swapping the linker domain from *P. falciparum* Top1 into the human enzyme replacing the 76 residues long human linker domain (from 636 to 712) with the 92 residues linker (from 695 to 787) from the *P. falciparum* enzyme. The hTop1(pf-Linker) has been found to have an extremely high religation rate and to be CPT resistant. A hTop1(pf-Linker), 3D model has been also generated and its structural dynamical properties have been investigated by molecular dynamics simulation. The results indicate that the pf-linker domain is sampling a much larger conformational space than the corresponding domain in the human enzyme confirming a strict correlation between linker dynamics and CPT reactivity.

## Materials and Methods

### Chemicals, Yeast strains and plasmids

Dymethyl sulfoxide (DMSO) and CPT were purchased from Sigma-Aldrich. CPT was dissolved in 99.9% DMSO to a final concentration of 4mg/ml (11.5 mM) and stored at -20°C.

ANTI-FLAG M2 monoclonal affinity gel, FLAG peptide and ANTI-FLAG M2 monoclonal antibody were purchased from Sigma-Aldrich.

*S. cerevisiae* top1 null strain EKY3 (*ura3-52, his3Δ200, leu2Δ1, trp1Δ63, top1::TRP1, MATα*) previously described [32], was used to express the hTop1 gene. YCpGAL1-e-hTop1 single copy plasmid was described previously [32,33]. The epitope-tagged construct, indicated as “e” contains the N-terminal sequence DYKDDDY and is recognized by the M2 monoclonal antibody.

The N-terminal flagged hTop1 1-635 and 713-765 fragments and *P. falciparum* Top1 695-787 linker domain from a synthetic gene codon optimized for expression in yeast [34] were PCR amplified using the “Expand high fidelity PCR system” purchased by Roche. To amplify the hTop1 fragments two couples of primers have been used: 5’-ATGAGTGGGGACCACCTCC-3’ and 5’-TGCCCTCTGATGGTTACAAAG-3’ for Top1 1-635 and 5’-CAGATTGCCCTGGGAACCTC-3’ and 5’-CTACTAAAACTCATAGTCTTCATCAGCC-3’. For the *P. falciparum* linker 695-787 two primers overlapping to each one of the Top1 fragments were designed: 5’-CTTTGTAACCATCAGAGGGCAATTCCAAAGCAACATGATACTACC-3’ and 5’-GAGGTTCCCAGGGCAATCTGTTTTGTTATCATCTCTAACCTTCATTTGATTG-3’. The three separate PCR reactions were ligated to obtain the full length domain-swapped product, hTop1(pf-Linker), comprising the *P. falciparum* linker domain embedded in the e-hTop1 N-terminal/Core/C-terminal domains. It has been added A-overhang to the obtained construct using a Taq DNA polymerase (Sigma-Aldrich) and then the specific 2.4 Kb band has been purified from 0.7% agarose gel in 1X TBE buffer (48 mM Tris, 45.5 mM boric acid, 1 mM EDTA) using the gel extraction kit QIAEX II purchased by Qiagen. The hTop1(pf-Linker), construct was cloned into pYES2.1/V5-His-TOPO expression vector (Invitrogen), according to the provided manufacturer protocol. The cloning reaction was transformed into XL10-Gold *E. coli* cells (Agilent Technologies), and a positive clone was identified by sequencing the extracted plasmid DNA.

### hTop1 and hTop1(pf-Linker) purification

hTop1 and hTop1(pf-Linker) were cloned in a single copy plasmid, YCpGAL1 and in pYES2.1/V5-His-TOPO, respectively under a galactose inducible promoter. They were transformed in top1 null EKY3 yeast strain using a lithium acetate procedure. Cells were grown overnight on SC-uracil plus 2% dextrose, at an optical density of A_595_=1.0 they were diluted 1:100 in SC-uracil plus 2% raffinose. At an optical density of A_595_=1.0, the cells were induced with 2% galactose for 6 h. Cells were then centrifuged, washed with cold water and resuspended in 2 ml buffer/g cells (50 mM Tris-HCl, pH 7.4, 1 mM EDTA, 1 mM EGTA, 10% glycerol and protease inhibitors cocktail from Roche, supplemented with 0.1 mg/ml sodium bisulfate, 0.8 mg/ml sodium fluoride, 1mM PMSF and 1mM DTT). After addition of 0.5 volumes of 425–600 mm diameter glass beads, the cells were disrupted by vortexing for 30 seconds alternating with 30 seconds on ice and then were centrifuged at 15000g for 30 minutes. For homogenous protein preparations, the whole extracts were applied to an ANTI-FLAG M2 affinity gel (Sigma-Aldrich) already equilibrated in according with the manufacturer protocol. Then columns were washed with 20 volumes of TBS (50 mM Tris–HCl pH 7.4 and 150 mM KCl) supplemented with the protease inhibitors, prior to load the lysate. Elution of e-hTop1, or e-hTop1(pf-Linker), was performed by competition with five column volumes of a solution containing 1mg of FLAG peptide (DTKDDDDK) in TBS. Fractions of 500 µl were collected and 40% glycerol was added in all preparations, which were stored at -20°C [23]. Protein levels and integrity were assessed by immunoblot with the monoclonal anti-FLAG M2 antibody (Sigma-Aldrich). The hTop1 and hTop1(pf-Linker) similar concentrated fractions were also compared to the purified Top1 with a known concentration (provided from Topogene) by immunoblot using the ab58313 Anti-Top1 antibody (Abcam) and ab97240 goat polyclonal Secondary Antibody (Abcam). The relative concentration of the two chosen fractions was estimated by a densitometry quantification using ImageJ software (NIH, http://rsb.info.nih.gov/ij/). The *in vitro* experiments have been performed using equal amount of purified hTop1 and hTop1(pf-Linker).

### DNA relaxation assays

The activity of 1 µl of hTop1 (12ng/µl) or hTop1(pf-Linker) (12ng/µl) was assayed in 30 µl of reaction volume containing 0.5 µg of negatively supercoiled pBlue-Script KSII(+) DNA, that is present in both dimeric and monomeric forms and reaction buffer (20 mM Tris–HCl pH 7.5, 0.1 mM Na _2_EDTA, 10 mM MgCl_2_, 50µg/ml acetylated bovine serum albumin and 150 mM KCl) [30,32].

The effect of CPT on enzyme activity was measured by adding DMSO or 100µM of the drug to the reactions, that were stopped with 0.5% SDS after each time-course point at 37°C. The samples were resolved in a 1% (w/v) agarose gel in 48 mM Tris, 45.5 mM boric acid, 1 mM EDTA at 10 V/cm. The gels were stained with ethidium bromide (0.5 µg/ml), destained with water and photographed using a UV transilluminator.

### Cleavage/Religation Equilibrium

Oligonucleotide CL25 (5'-GAAAAAAGACTTAGAAAAATTTTTA-3') that contains a hTop1 high affinity cleavage site, was 5´-end labelled with [γ32P] ATP. The CP25 complementary strand (5'-TAAAAATTTTTCTAAGTCTTTTTTC-3') was 5´-end phosphorylated with unlabelled ATP. The two strands were annealed with a 2-fold molar excess of CP25 over CL25 [35]. A final concentration of 20 nM duplex CL25/CP25 was incubated with an excess of hTop1 or hTop1(pf-Linker) enzymes (0.3 µg) at 25°C in 10 mM Tris-HCl pH 7.5, 5 mM MgCl_2_, 5 mM CaCl_2_, 150 mM KCl in the presence or absence of 100 μM CPT. At different time points, the reactions were stopped by adding 0.5% SDS, after ethanol precipitation samples were resuspended in 5 μl of 1 mg/ml trypsin to remove the protein and were analyzed by denaturing 7M urea/ 16% polyacrylamide gel electrophoresis in 48 mM Tris, 45.5 mM Boric Acid, 1 mM EDTA. The percentage of the stabilized cleavable-complex was determined by PhosphorImager and ImageQuant software and normalized on the total amount of radioactivity in each lane.

### Cleavage kinetics

Oligonucleotide CL14 (5'-GAAAAAAGACTTAG-3') that contain a hTop1 high affinity cleavage site, was 5´-end labelled with [γ32P] ATP. The CP25 complementary strand (5'-TAAAAATTTTTCTAAGTCTTTTTTC-3') was 5´-end phosphorylated with unlabeled ATP. CL14/CP25 was annealed as previously described in the cleavage/religation equilibrium experiment. The suicide cleavage reactions were carried out by incubating 20 nM of the duplex DNA with an excess (0.36 µg) of hTop1 or hTop1(pf-Linker) enzymes in 10 mM Tris pH 7.5, 5 mM MgCl_2_, 5 mM CaCl_2_ and 150 mM KCl at 25°C in a final volume of 60 μl [36]. At various time points 5 μl aliquots were removed and the reaction stopped with 0.5% (w/v) SDS. After ethanol precipitation samples were resuspended in 5 μl of 1 mg/ml trypsin and incubated at 37°C for 60 minutes. However a short trypsin resistant peptide is always left explaining why the Cl1 migrates slower than the Cl14 oligonucleotide [12]. Samples have been analyzed by denaturing 7 M urea/20% polyacrylamide gel electrophoresis in TBE (48 mM Tris, 45.5 mM Boric Acid, 1 mM EDTA).

Oligonucleotide CL14-U (5'-GAAAAAAGACTUAG-3') was 5’ end labelled and annealed with CP25 as for the CL14. 20 nM substrate has been incubated with an excess (0.24 µg) of hTop1 or hTop1(pf-Linker) enzymes in 10 mM Tris pH 7.5, 5 mM MgCl_2_, 5 mM CaCl_2_ and 150 mM KCl at 25°C in a final volume of 40 μl. At various time points 5 μl aliquots were removed and the reaction stopped with 0.5% (w/v) SDS and directly loaded without ethanol precipitation and trypsin digestion. Samples have been analyzed by denaturing 7 M urea/20% polyacrylamide gel electrophoresis in TBE (48 mM Tris, 45.5 mM Boric Acid, 1 mM EDTA). In both experiments the percentage of cleaved substrate (Cl1) was determined by PhosphorImager and ImageQuant software and normalized on the total amount of radioactivity in each lane.

### Religation kinetics

20 nM of CL14/CP25 (radiolabeled as previously described in the cleavage kinetic experiment) was incubated with an excess (0.36 µg) of hTop1 or hTop1(pf-Linker) for 60 minutes at 25°C followed by 30 minutes at 37°C in 20 mM Tris-HCl pH 7.5, 0.1 mM Na _2_EDTA, 10 mM MgCl_2_, 50 μg/ml acetylated BSA, and 150 mM KCl. After the formation of the cleavable complex (Cl1) a 5µl aliquote was removed and used as time 0 point, then DMSO or 100 µM CPT were added and religation reaction was started by adding a 200-fold molar excess of R11 oligonucleotide (5'-AGAAAAATTTT-3') over the CL14/CP25 [12]. 5 μl aliquots were removed at various time points, and the reaction stopped with 0.5% SDS. After ethanol precipitation, samples were resuspended in 5 μl of 1 mg/ml trypsin and incubated at 37°C for 60 minutes. Samples were analyzed by denaturing 7 M urea/20% polyacrylamide gel electrophoresis in 48 mM Tris, 45.5 mM Boric Acid, 1 mM EDTA. The religation percentage was quantified by ImageQuant software then the ratio between the religation band and the total radioactivity for each lane was plotted as the time function and normalized to the plateau value.

### Generation of the Model

The amino acid sequence for the pfTop1 with code **Q26024** was taken from the server www.expasy.org/sprot. The sequence was first aligned against the entire protein sequence databank using the BLAST tool [37]. The best aligned sequence for which the 3D structure is known resulted to be the hTop1 whose entry for the swissprot server is **P11387**. The pair wise alignment was then refined using the program T-COFFEE [38].

Based on the sequence alignment, the template considered for the homology modeling was the crystal structures **1A36** of the hTop1 in complex with a 22 bp DNA [6]. The residues of the protein present in the crystal structure range from Gln201 to Phe765 and the corresponding pfTop1 residues, based on the alignment, range from Pro140 to Phe839. The modeling has been done using the automatic server SwissModel [39,40], using the Alignment Mode. The model has been checked for clashes and wrong contacts with the Procheck validation server (http://www.ebi.ac.uk/thornton-srv/software/PROCHECK/) and the wrong bonds and angles have been regularized using the program Sybyl v. 6.0 (TRIPOS, http://www.tripos.com/), performing an energy minimization with the Powell Method [41].

Once the model has been obtained, the linker of the PfTop1 protein spanning between residues Ile695–Lys787, has been substituted to the human linker formed by residues Pro636–Lys712, so that the new linker is formed by residues Ile636–Lys728, being 16 residues longer (Figure S1). The added residues are found at the tip of the linker, in the coil region connecting the two helices that upon this introduction is now formed by a coil-helix-coil motif (Figure S1). Due to the introduction of the PfTop1 linker, the number of the catalytic tyrosine is now 739.

### Molecular Dynamics

Molecular Dynamics simulation has been carried out on the covalent hTop1(pf-Linker)-DNA complex upon inserting a DNA substrate into the protein model. The structure of the core and C-terminal domains of the hTop1 and the hTop1(pf-Linker) have been structurally aligned to model the DNA substrate coming from the covalent enzyme DNA complex from the **1K4S** crystal structure [21] using the program SwissPdbViewer [42]. The structure was then minimized with the Powell minimization algorithm [41] implemented in the Sybyl 6.0 program (TRIPOS, http://www.tripos.com/). The protein-DNA complex was parameterized with the AMBER03 all-atoms force field [43] implemented in the GROMACS MD package 4.0.7 [44] from Sorin and Pande [45]. The complex was placed in a rectangular box (109x160x120 Å^3^), filled with TIP3P water molecules [46] and rendered electroneutral by the addition of 24 Na^+^ counterions, for a total of 215294 atoms. Electrostatic interactions have been taken into account by means of the Particle Mesh Ewald method [47] and the SHAKE algorithm [48] has been used to apply a constraint on all hydrogen bonds length. Optimization and relaxation of solvent and ions were initially performed keeping the protein-DNA atoms constrained to their initial position with decreasing force constants of 1000 and 500 kJ/(mol • nm), for 500 ps. The system has then been simulated for 75 ns at a constant temperature of 300 K using the Berendsen’s method [49] and at a constant pressure of one bar; the pressure was kept constant (1 bar) using the Rahman-Parrinello barostat [50] with a 2.0 fs time step. All the analyses have been performed with the GROMACS MD package v. 4.0.7 [44], images were obtained with the VMD program [51] and graphs with the Grace program.

The analyses have been performed considering the last 72 ns of simulation, once eliminated the equilibration time, and have been compared to a previously performed simulation of the human enzyme [20].

## Results

### Relaxation assays

**Figure 1:**
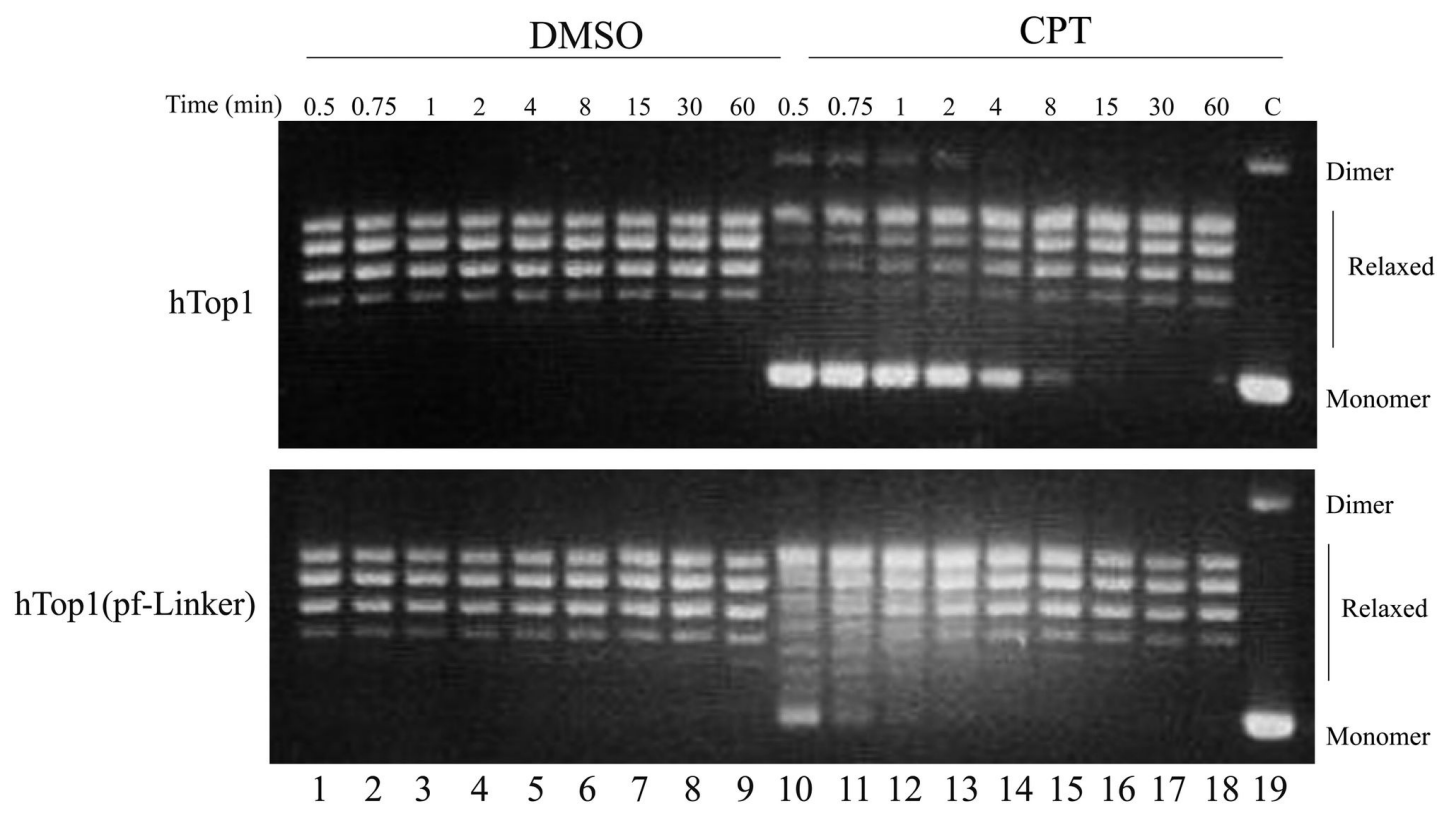
Relaxation of supercoiled DNA. Relaxation of negative supercoiled plasmid in a time course experiment for hTop1 and hTop1(pf-Linker) in presence of DMSO (lanes 1–9) and 100µM CPT (lanes 10–18); lane 19, no protein added. The reaction products are resolved in an agarose gel and visualized with ethidium bromide. The two forms of the supercoiled plasmid DNA are indicated as "Dimer" and "Monomer".

The relaxation activity of both hTop1 and hTop1(pf-Linker) has been assessed incubating 12 ng of each enzyme with 0.5µg of a negative supercoiled plasmid in the presence or absence of 100 µM CPT. In the absence of the drug the same concentration of DMSO used in its presence was added in order to directly compare the experiments. The reactions were stopped after different times interval and the products resolved by agarose gel electrophoresis. As shown in Figure 1, the two enzymes exhibit comparable relaxation activities, since they completely relax the supercoiled DNA after 0,5 minute of incubation (Figure 1, lanes 1-9). The presence of CPT considerably slows down the relaxation by hTop1 and a full relaxation is observed after about 15 minutes from the drug addition (Figure 1, lane 16) [12,52]. On the other hand CPT has a moderate effect on the hTop1(pf-Linker), relaxation since a full relaxation can be observed after 1-2 minutes of the drug addition (Figure 1, lane 12-13). A relaxation assay has been carried out also using half of the enzyme concentration. In the absence of the drug the two enzymes have an identical relaxation rate (Figure S2, lanes 1-9), upon addition of CPT hTop1 relaxes the DNA after 15 minutes (Figure S2, lane 17) while hTop1(pf-Linker) after 5 minutes (Figure S2, lane 16) confirming its low CPT sensitivity. This result suggests that CPT cannot efficiently stabilize the hTop1(pf-Linker)-DNA complex and that the substitution of the human linker domain with the corresponding domain from the *P. falciparum* enzyme confers CPT resistance to the enzyme.

### Cleavage/religation equilibrium analysis

**Figure 2:**
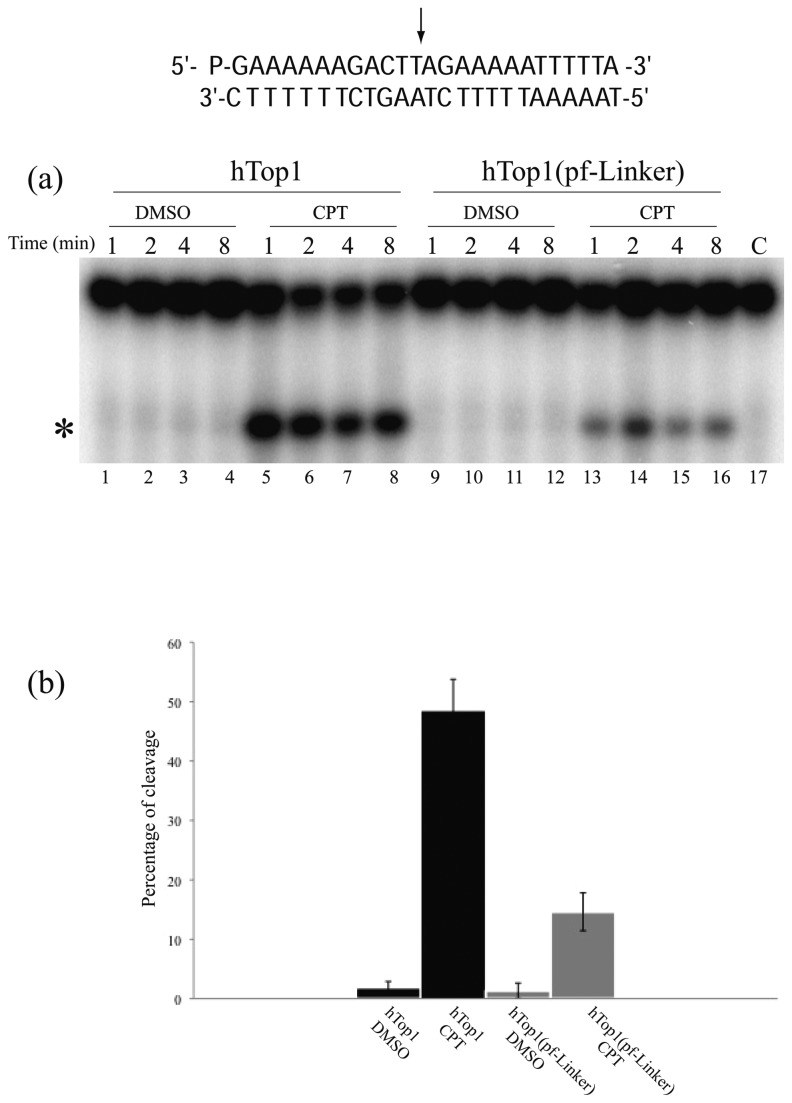
Cleavage/Religation Equilibrium. (a) Gel electrophoresis of the products generated by incubation of hTop1 (lanes 1-8) and hTop1(pf-Linker) (lanes 9-16) with the [γ -^32^P] end-labelled duplex DNA (CL25/CP25), shown at the top of the figure. The arrow indicates the preferred cleavage site. The duplex was incubated for different time intervals with hTop1 in absence (lanes 1-4) and in presence of CPT (lanes 4-8) or with hTop1(pf-Linker) in absence (lanes 9-12) and in presence of CPT (lanes 13-16). Lane 17, no enzyme added. The band corresponding to the enzyme–substrate cleaved complex, has been indicated by an asterisk. (b) Percentages of the hTop1 (black) or hTop1(pf-Linker) (grey) cleavage complex generated after 1 minute of incubation normalized to the total amount of radiolabeled DNA in each lane. Data shown are means ± SD from 3 independent experiments.

The stability of the covalent DNA-enzyme complex was analyzed by incubating purified hTop1 or hTop1(pf-Linker) with a 25 bp fully duplex oligonucleotide substrate CL25/CP25 in the presence or absence of CPT for different time intervals (Figure 2). Incubation of the hTop1(pf-Linker) with the substrate in absence of the drug (Figure 2a, lanes 9-12) gives rise to a weak band with a mobility corresponding to the mobility of the cleavage product produced by hTop1 and with an intensity comparable of the one observed for the human enzyme (Figure 2a, lanes 1-4). In presence of CPT the hTop1 cleavage is strongly increased (Figure 2a, lanes 5-8), while the effect is considerably less pronounced in the case of the hTop1(pf-Linker) (Figure 2a, lanes 13-16). This difference can be well appreciated from the graph in Figure 2b, where the histogram depicts the percentages of stabilized cleavage products detected after 1 minute of incubation relative to the total amount of radiolabeled DNA in each lane. The relatively low intensity of the band representing cleavage-products generated by the hTop1(pf-Linker) (Figure 2a, lanes 13-16) in the presence of CPT confirms the partial CPT resistance of hTop1(pf-Linker). In order to identify the steps of the enzyme catalytic cycle that are influenced by the presence of the long plasmodial linker domain, the cleavage and religation steps of catalysis were analyzed separately.

### Cleavage and religation kinetics

**Figure 3:**
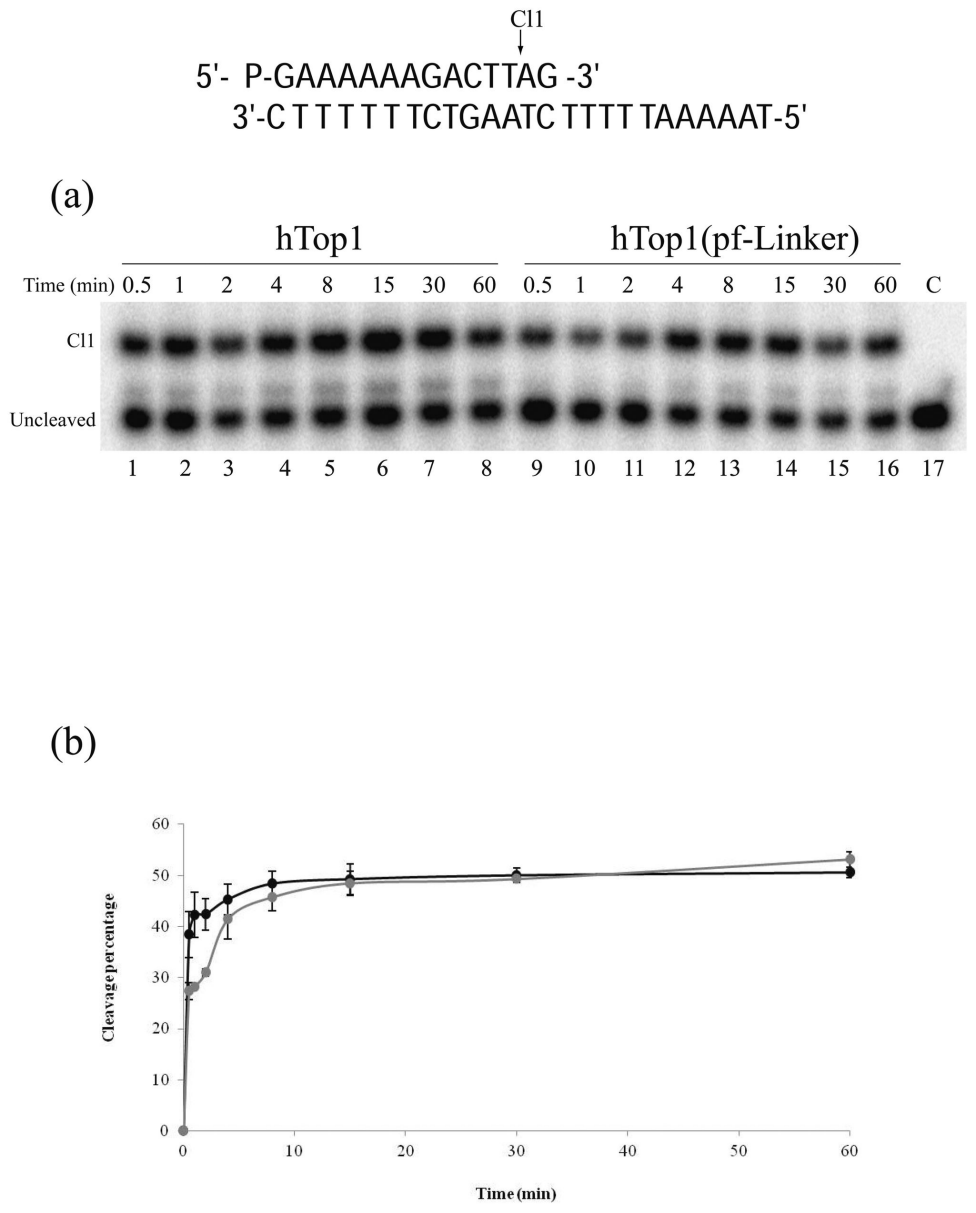
Cleavage Kinetics using suicide substrate. (a) Time course (0.5-60 minutes) of the cleavage reaction of the purified hTop1 (lanes 1-8), or hTop1(pf-Linker) (lanes 9-16) with the CL14/CP25 suicide substrate, shown at the top of the figure. In lane 17 the protein has not been added. Cl1 represents the DNA substrate cleaved by the enzymes at the preferred cleavage site. (b) Percentage of cleaved suicide substrate plotted against time for the reaction with Top1 (black) and with hTop1(pf-Linker) (grey). Data shown are means ± SD from 3 independent experiments.

**Figure 4:**
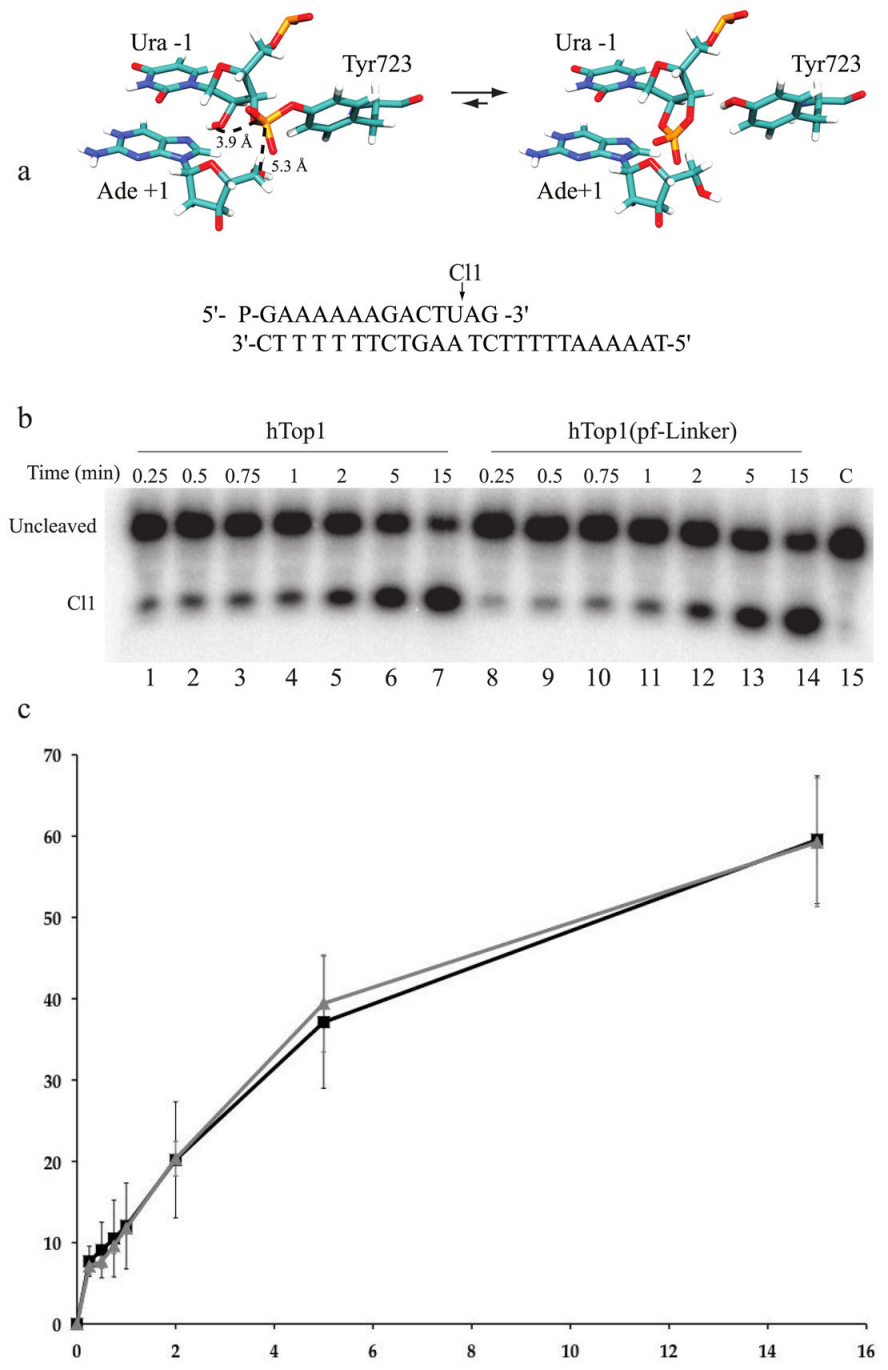
Cleavage Kinetics using ribo modified substrate. (a) Sequence of the CL14-U/CP25 substrate and highlight of the Top1 cleavage site containing a scissile ribonucleoside monophosphate. (b) Time course (0.25-15 minutes) of the cleavage reaction of purified hTop1 (lanes 1-8), or hTop1(pf-Linker) (lanes 9-16) with the CL14-U/CP25 substrate (shown in a). In lane 15 the protein has not been added. Cl1 represents the DNA substrate cleaved by the enzymes at the preferred cleavage site. (c) Percentage of cleaved substrate plotted against time for the reaction with hTop1 (black) and with hTop1(pf-Linker) (grey). Data shown are means ± SD from 3 independent experiments.

The cleavage rates of the two enzymes were compared in a time course experiment using a CL14/CP25 suicide DNA substratewhere the preferential hTop1 cleavage site is indicated by an arrow in the top panel of Figure 3a. After the cleavage reaction, the enzyme remains covalently attached to the 3’ end of the cleaved strand, being unable to religate the dinucleotide AG that is cleaved off and diffuses away. Following the incubation of 20 nM suicide substrate with 0.36 µg of either hTop1 or hTop1(pf-Linker), at increasing time intervals, the reaction products were EtOH precipitated and trypsinated before denaturing PAGE analyses. Due to the covalent attachment of a short trypsin resistant peptide the cleavage products have a slower mobility than the substrate in PAGE as previously demonstrated [53]. As evident from Figure 3a, hTop1 (lanes 1-8) and hTop1(pf-Linker) (lanes 9-16) exhibit a comparable rate of cleavage on the suicide DNA substrate. A quantitative analysis, obtained by plotting the percentage of cleavage products, normalized to the total amount of radiolabeled DNA in each lane (Figure 4b), confirm that the cleavage rates of the two enzymes are identical.

To better confirm this result the cleavage rate of both hTop1 and hTop1(pf-Linker) has been addressed also using a CL14-U (5’-GAAAAAAGACTUAG-3’) oligonucleotide, containing an uracil at the preferred Top1 cleavage site [54]. This oligonucleotide, having the deoxyribo-thymine (dT) in position 12 substituted by a ribo-Uracil (rU), has been annealed to the CP25 (5’-TAAAAATTTTTCTAAGTCTTTTTTC-3’) complementary strand, to produce a duplex with an 11-base 5’-single-strand extension. With this oligonucleotide the enzyme does not remain trapped over the substrate since, after cutting at the preferential site, the 2’-OH of the ribose attacks the 3’-phosphotyrosyl linkage between the enzyme and the ribonucleotide, releasing the enzyme and leaving a 2’,3’-cyclic phosphate end (Figure 4a). In this way possible artifacts due to the religation of the short dinucleotide-AG are avoided and the cleavage rate can be better evaluated. The same concentration of hTop1 and hTop1(pf-Linker) has been incubated with this substrate and the fragments have been resolved in a time course experiment in a denaturing polyacrylamide gel reported in Figure 4b, where the Cl1 cleaved oligonucleotide runs like a 12 mer since it does not have any protein covalently bound to it. Analysis of the Cl1 intensity band unambiguously demonstrates that hTop1 and hTop1(pf-Linker) have an identical cleavage rate and they reach the same plateau value.

**Figure 5:**
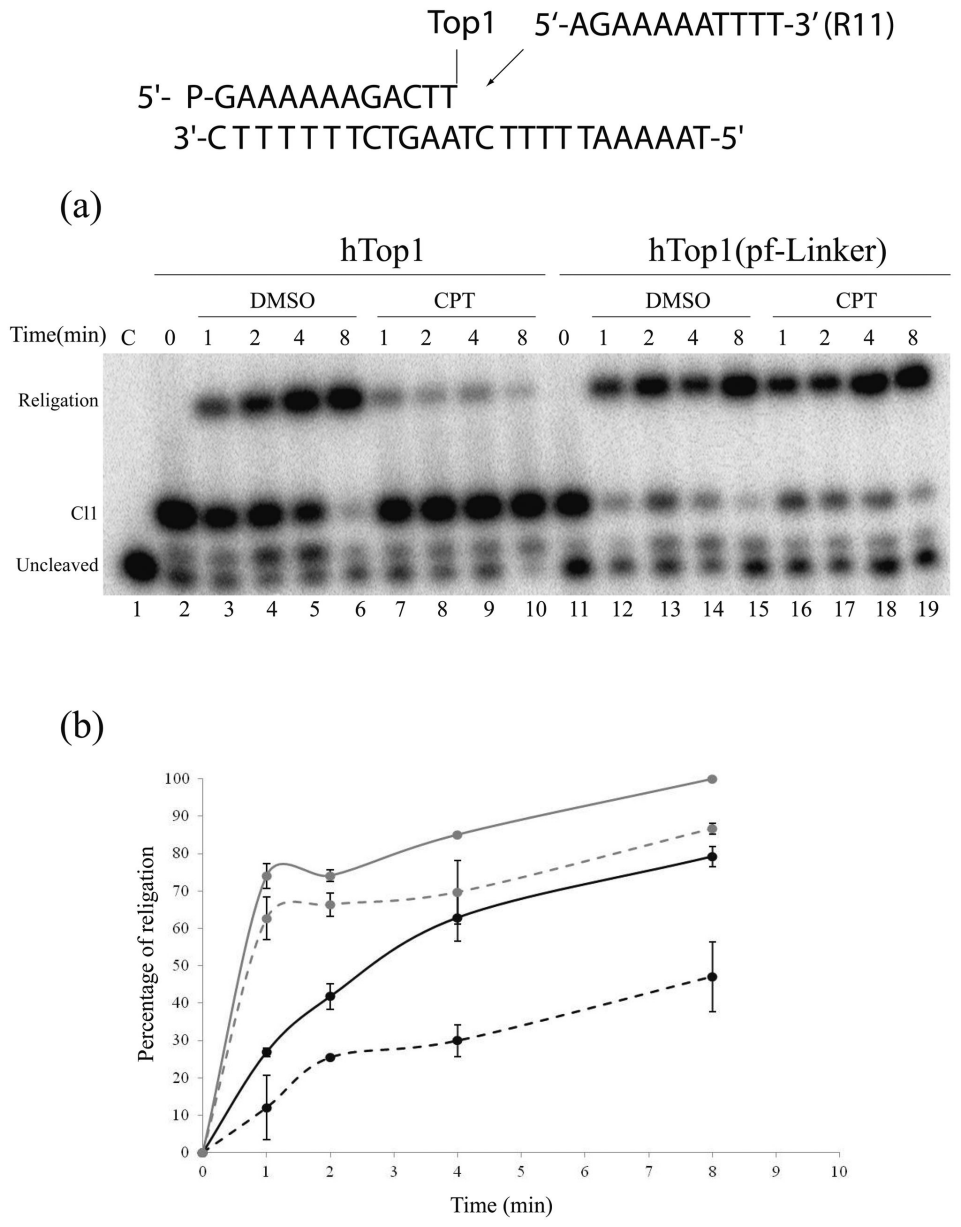
Religation Kinetics. (a) Gel analysis of the religation kinetics observed when incubating the hTop1- or hTop1(pf-Linker)-suicide cleavage complex (Cl1) with the R11 complementary ligator oligonucleotide (shown at the top of the figure) in absence [lanes 3-6 and lanes 12-15 for hTop1 and hTop1(pf-Linker) respectively] or in presence of 100 µM CPT [lanes 7-10 for hTop1 and lanes 16-19 for hTop1(pf-Linker)]. In lane 1 no protein was added. The lanes 2 and 11 represent the time 0 for hTop1 and hTop1(pf-Linker) mediated reactions, respectively. “Cl1” represents the DNA fragment cleaved at the preferred enzyme site; “religation” is the restored fully duplex oligonucleotide representing the final product of the religation reaction. (b) Plot of the percentage of religation normalized to the plateau value, in absence or in presence of CPT for the hTop1 (full and dashed black lines, respectively) and hTop1(pf-Linker) (full and dashed grey lines, respectively). Data shown are means ± SD from 3 independent experiments.

The DNA religation kinetics of hTop1 and hTop1(pf-Linker) were compared incubating 0.36 µg of hTop1 or hTop1(pf-Linker) enzymes with 20 nM the suicide substrate CL14/CP25 for 60 minutes at 25°C and for other subsequent 30 minutes at 37°C. After removing an aliquot of the reaction, representing time 0 (Figure 5a, lanes 2 and 11), the R11 ligator oligonucleotide (see upper panel of Figure 4a), having a sequence complementary to the 11-base 5’ overhang of the generated cleavage complexes was added to initiate religation and incubation were continued for different time intervals in the presence or absence of CPT at 37°C. Following termination of the reactions by the addition of SDS, the samples were EtOH precipitated, trypsin digested and analyzed by denaturing PAGE (Figure 5a). The hTop1(pf-Linker) religates the complementary strand considerably faster than hTop1 (compare lanes 3-6 with lanes 12-15 in Figure 5a). The presence of CPT strongly inhibits the religation reaction mediated by hTop1 (Figure 5a, lanes 7-10)(10,50), while the drug has little or no effect on hTop1(pf-Linker) (Figure 5a, lanes 16-19). The extent of religation was quantitatively evaluated plotting the religation products as a function of time (Figure 5b). The result indicates that the religation percentage relative to hTop1(pf-Linker) is always higher than hTop1 at any time point and that this quantity is slightly affected by the presence of CPT, definitely demonstrating that hTop1(pf-Linker) is CPT resistant, due to the inability of CPT to stabilize the hTop1(pf-Linker)-substrate cleavage complex.

### Molecular dynamics simulation of the hTop1(pf-Linker) model

**Figure 6:**
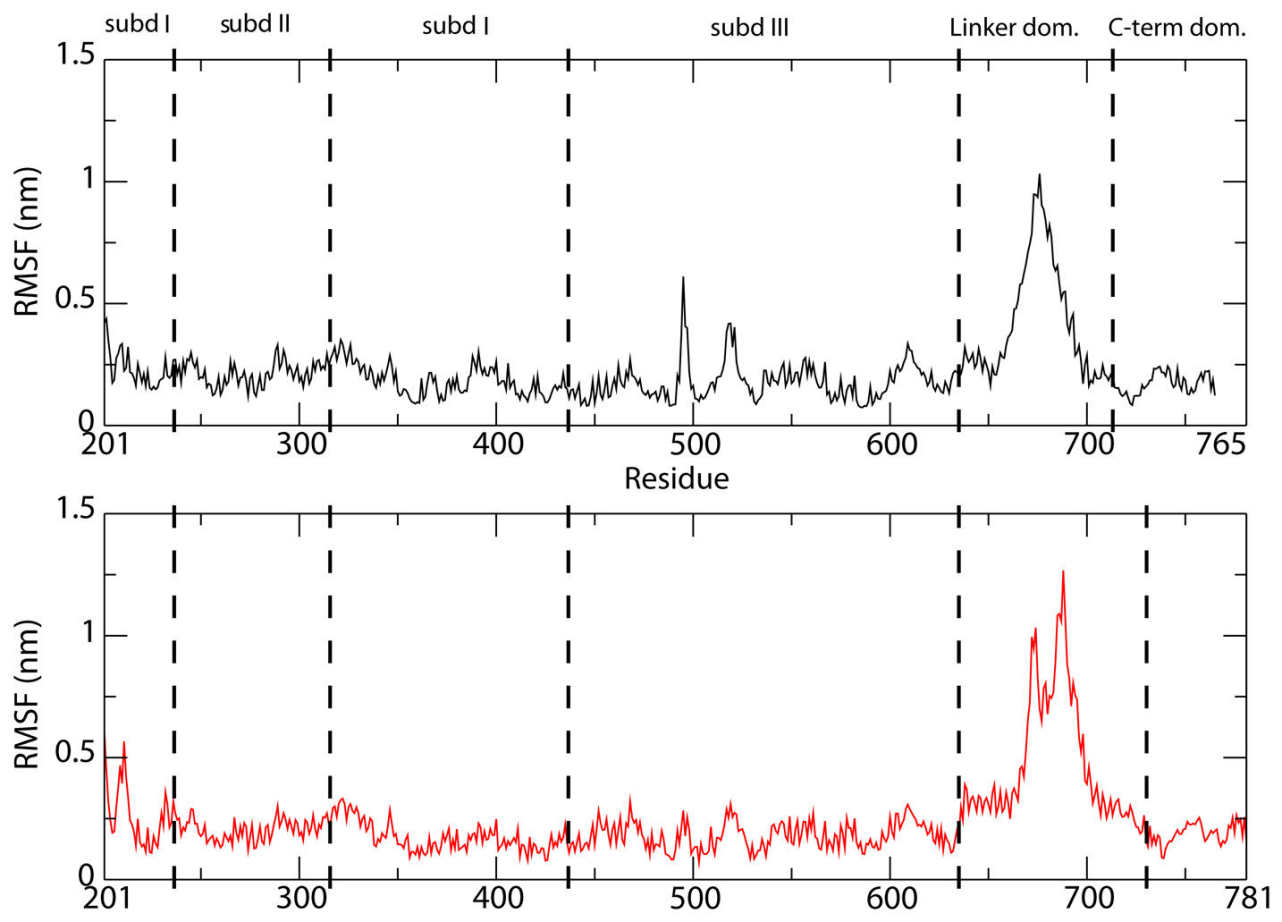
Root Mean Square Fluctuation. Per-residue Root Mean Square Fluctuations (RMSF) of the hTop1, upper panel black line, and of the hTop1(pf-Linker) model, lower panel red line. In both panels the border of the protein domains is reported.

The structural-dynamical effect of the substitution of the linker in the hTop1 with the plasmodial counterpart has been investigated by molecular dynamics simulation of a hTop1(pf-Linker) model (Figure S1), built as described in Material and Methods. The simulation of the protein in covalent complex with a 22 bp DNA double strand shows that the plasmodial linker displays a larger flexibility than the human linker domain, as evidenced by the RMSF analysis (Figure 6). The core and C-terminal domain residues have on average a comparable flexibility. The larger flexibility of the hTop1(pf-Linker) can be appreciated also by the RMSD values that are larger than the hTop1 enzyme (Figure S3, black and red lines), although for both proteins, the value of the RMSD calculated as a function of time decreases upon elimination of the contribution of the linker domain (Figure S3, green and blue lines).

**Figure 7:**
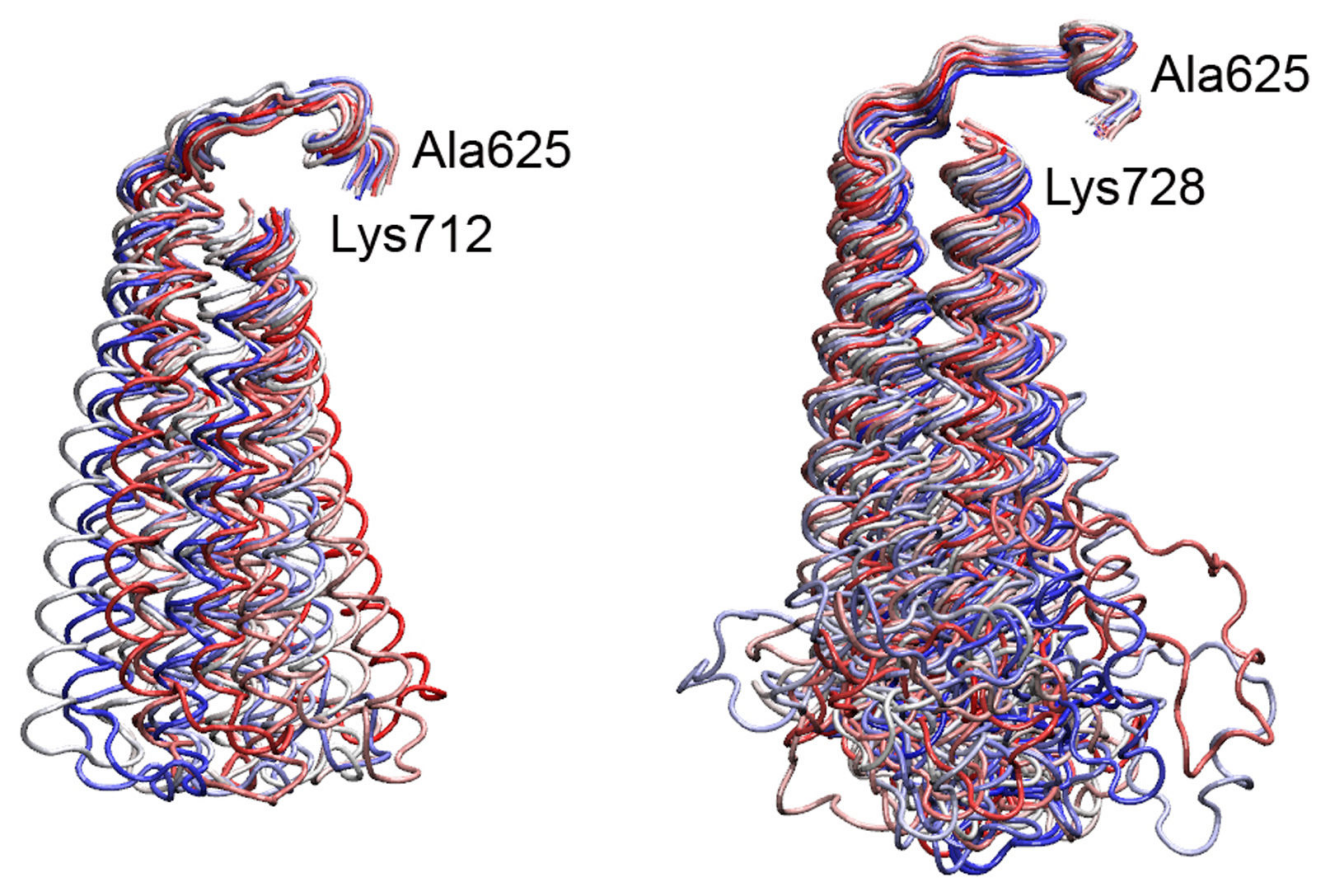
Cluster analysis of the linker. Centroids of the clusters representing the families of the linker domain structures for hTop1, left panel, and for the hTop1(pf-Linker), right panel. The structures are colored following the color code blue-white-red, from the most to the less populated cluster.

**Figure 8:**
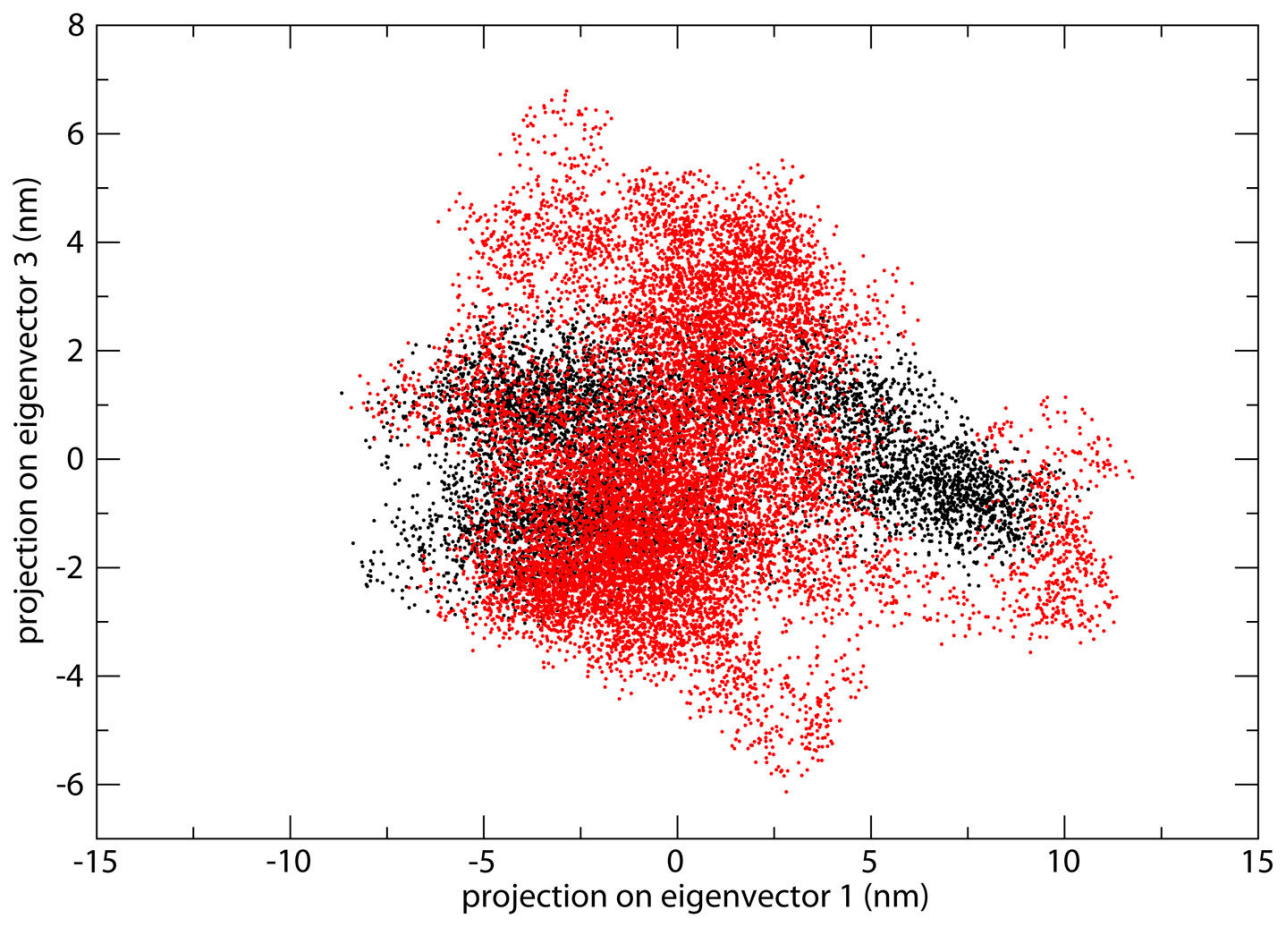
Principal Components Analysis. Projection of the motion along the planes formed by the eigenvectors 1 and 3. The data for the hTop1 and hTop1(pf-Linker) proteins are reported in black and red, respectively.

The ability of the linker domain to sample a larger conformational space in the hTop1(pf-Linker) than in the hTop1 enzyme is confirmed by a cluster analysis (Figure 7). Overlapping and minimizing the different frames of the simulation over the core and C-terminal domains Cα atoms gives rise to 43 clusters for the linker in hTop1(pf-Linker) and to 14 clusters for the human protein. In detail, 90% of the total clustered structures are represented by the first 7 and the first 2 families for the hTop1(pf-Linker) and hTop1, respectively. This behavior can also be appreciated by analyzing the principal components that describe the motion of the proteins along the simulations. In the case of the hTop1, 80% of the total motion is described by the first 4 eigenvectors, while in the case of the hTop1(pf-Linker) it is described by the first 8 eigenvectors (Figure S4), indicating that the amplitude of the motion in the hTop1(pf-Linker) is spread along a wider conformational space. This feature can be further monitored by the plot of the projection of the Cα atoms trajectories on the planes formed by the first and third eigenvectors (Figure 8), indicating that in the hTop1 the motion is more confined when compared to the hTop1(pf-Linker) enzyme. This is most evident for what concerns the eigenvector 3 (Figure 8), along which the amplitude of motion for the hTop1 and hTop1(pf-Linker) enzyme is 64 Å and 127 Å respectively (Figure 8).

In line, the plot of the RMSF calculated along the single eigenvectors (Figure S5) indicates that in the hTop1, the motion of the linker is mainly described by the first eigenvector, while in the case of the hTop1(pf-Linker) the motion of the domain is described by the first three eigenvectors, confirming that the motion of the linker in the hTop1(pf-Linker) is spread along several directions (Figure S5)

## Discussion

The presented results demonstrate that the replacement of the hTop1 linker domain with the corresponding domain from *P. falciparum* alters the enzyme function and drug reactivity *in vitro*. The hTop1(pf-Linker) enzyme displays the same relaxation activity and cleavage rate as the hTop1 enzyme (Figure 1 Figures S2, 3 and 4). However, hTop1(pf-Linker) is considerably less sensitive to CPT during relaxation than hTop1 (Figure 1 Figure S2). The partial CPT resistance of the hTop1(pf-Linker) is confirmed by the cleavage/religation equilibrium experiment (Figure 2), where the band corresponding to the CPT stabilized enzyme-DNA cleavage complex has a much lower intensity for hTop1(pf-Linker) than for hTop1. A comparative analysis of the religation rate provides an explanation for the CPT resistance displayed by the hTop1(pf-Linker). The chimeric enzyme is in fact characterized by a faster religation rate when compared to hTop1 and its ligation is almost unaffected by the presence of CPT (Figure 5). Insertion of the *P. falciparum* linker in the human enzyme has then an important functional effect that is an increased religation rate accompanied by CPT insensitivity. The importance of the linker in modulating the enzyme function has been firstly demonstrated studying the enzyme deleted of the linker, that displays an increase of the religation rate and a decrease in CPT sensitivity [12]. The authors correctly concluded that the presence of the linker in the intact human enzyme, under normal circumstances, acts to modulate the religation and its loss brings to an increased religation rate. In this work we show that not only the loss of the linker but also the presence of a linker having a disabled flexibility, brings to an increased religation rate and a CPT insensitivity. In fact, analysis of the structural-dynamical properties of the hTop1(pf-Linker) model obtained by molecular dynamics simulation indicates that hTop1(pf-Linker) displays a conformational flexibility larger than the human enzyme, as evidenced by the increased RMSF (Figure 6) and samples a larger conformational space, as evidenced by the cluster analysis of the structures extracted from the trajectory (Figure 7). Consistently, the motion of the linker in the human enzyme is mainly described by the first eigenvector, while in the hTop1(pf-Linker) the same motion is spread along the first three eigenvectors, confirming that the linker dynamics in the hTop1(pf-Linker) is less confined than in the human enzyme (Figure 8 and Figure S5).

An identical correlation between linker conformational variability, fast religation rate and CPT insensitivity has previously been reported for a hTop1 mutant carrying the single mutation Ala653Pro on the linker domain [16]. Other papers have further demonstrated through experimental and computational analysis that a variability of linker flexibility results in a cleavage-religation equilibrium shifted toward religation and CPT resistance [13,24,25]. This is consistent with the fact that the mutants Lys681Ala and Asp677Gly-Val703Ile, that displays a reduced linker flexibility, have a reduced religation rate and an increased drug sensitivity [18,23].

In line with these findings, our study demonstrates that introduction of the long plasmodial linker domain into the human enzyme causes an increased religation rate and CPT insensitivity and opens the way of a systematic investigation of the correlation between the linker and the other protein domains to be exploited to identify species selective drugs.

## Supporting Information

Figure S1 Structure of the hTop1(**pf-Linker**) model. Side and upper view of the DNA-hTop1(pf-Linker) model covalent complex. The core and C-terminal domains belonging to the hTop1 are reported in red, and the linker coming from the *P. falciparum* protein is reported in lime.
(TIF)

Figure S2 Supercoiled DNA relaxation kinetics. Relaxation of negative supercoiled plasmid in a time course experiment for hTop1 and hTop1(pf-Linker) in presence of DMSO (lanes 1–9) and 100µM CPT (lanes 10–18); lane 19, no protein added. The reaction products are resolved in an agarose gel and visualized with ethidium bromide. The two forms of the supercoiled plasmid DNA are indicated as “Dimer” and “Monomer”..
(TIF)

Figure S3 Root Mean Square Deviation. Cα atoms Root Mean Square Deviation (RMSD) calculated as a function of time for the hTop1 with (black line) and without the linker domain (green line), for the hTop1(pf-Linker) with (red line) and without the linker domain (blue line).
(TIF)

Figure S4 Cumulative Percentage of Eigenvectors. Cumulative percentage of motion as a function of eigenvectors for the Cα atoms of the hTop1 (black line) and hTop1(pf-Linker) complex (red line).
(TIF)

Figure S5 Root Mean Square Fluctuation along Eigenvectors. Root Mean Square Fluctuations along the first three eigenvectors for the hTop1 (left panel, black line) and hTop1(pf-Linker) (right panel, red line) enzyme.
(TIF)
