# Supplementary figures and images for: Replacement of the Human Topoisomerase Linker Domain with the Plasmodial Counterpart Renders the Enzyme Camptothecin Resistant
Source: PLoS One. 2013 Jul 2;8(7):e68404. doi: 10.1371/journal.pone.0068404 (PMC3699648; doi:10.1371/journal.pone.0068404)

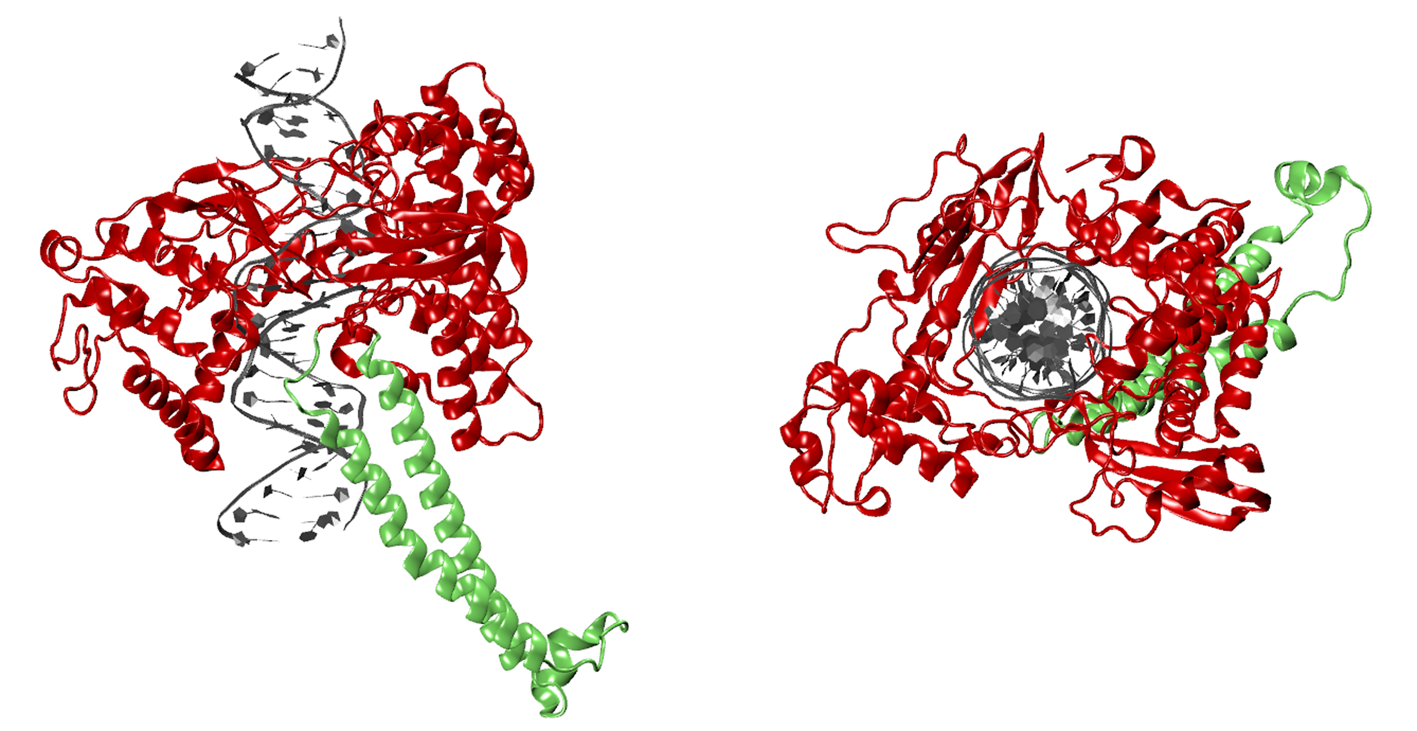

Supplement: Figure S1 — Side and upper view of the DNA-hTop1(pf-Linker) model covalent complex. The core and C-terminal domains belonging to the hTop1 are reported in red, and the linker coming from the P. falciparum protein is reported in lime. (TIF) [file pone.0068404.s001.tif]

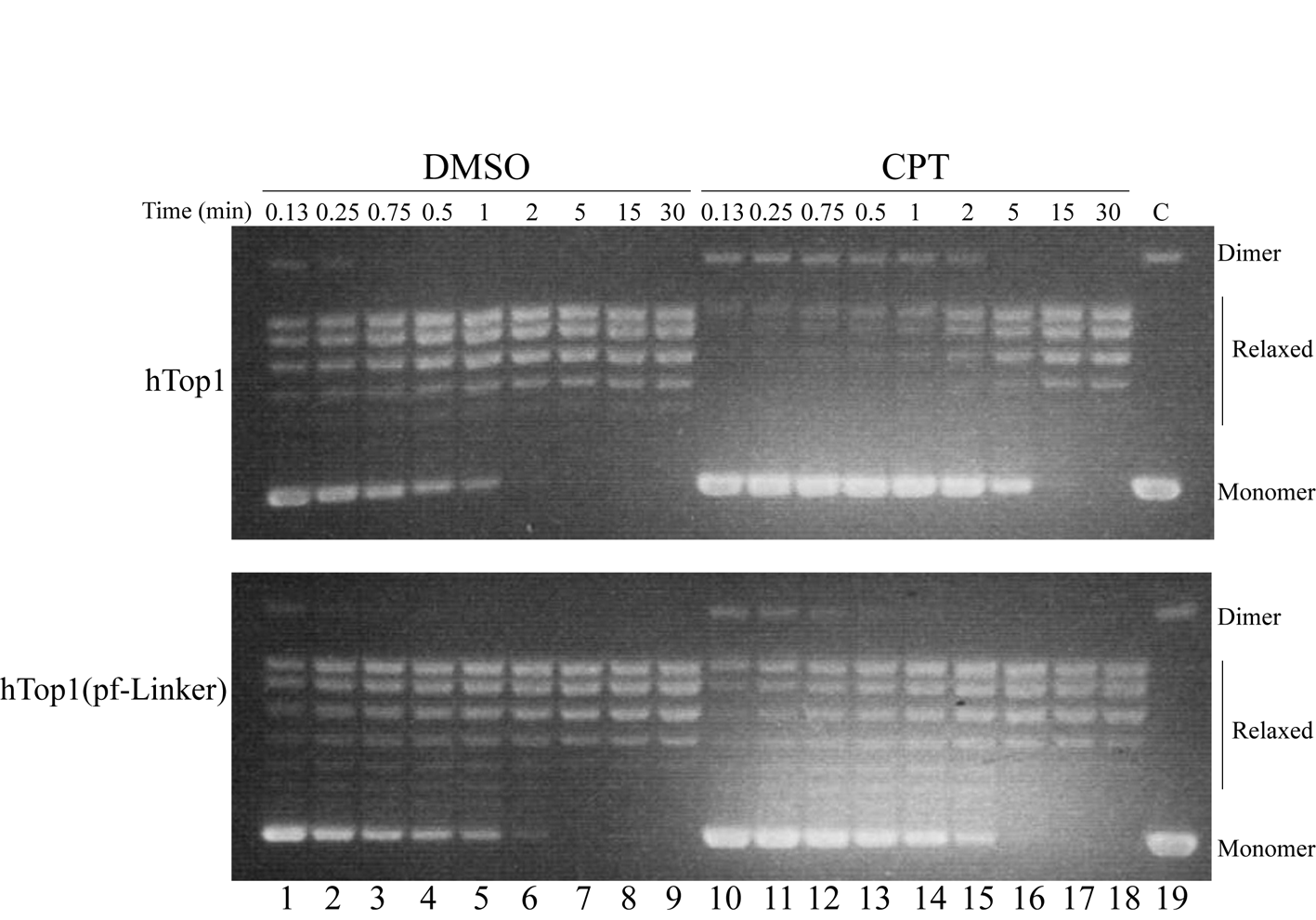

Supplement: Figure S2 — Relaxation of negative supercoiled plasmid in a time course experiment for hTop1 and hTop1(pf-Linker) in presence of DMSO (lanes 1–9) and 100µM CPT (lanes 10–18); lane 19, no protein added. The reaction products are resolved in an agarose gel and visualized with ethidium bromide. The two forms of the supercoiled plasmid DNA are indicated as “Dimer” and “Monomer”.. (TIF) [file pone.0068404.s002.tif]

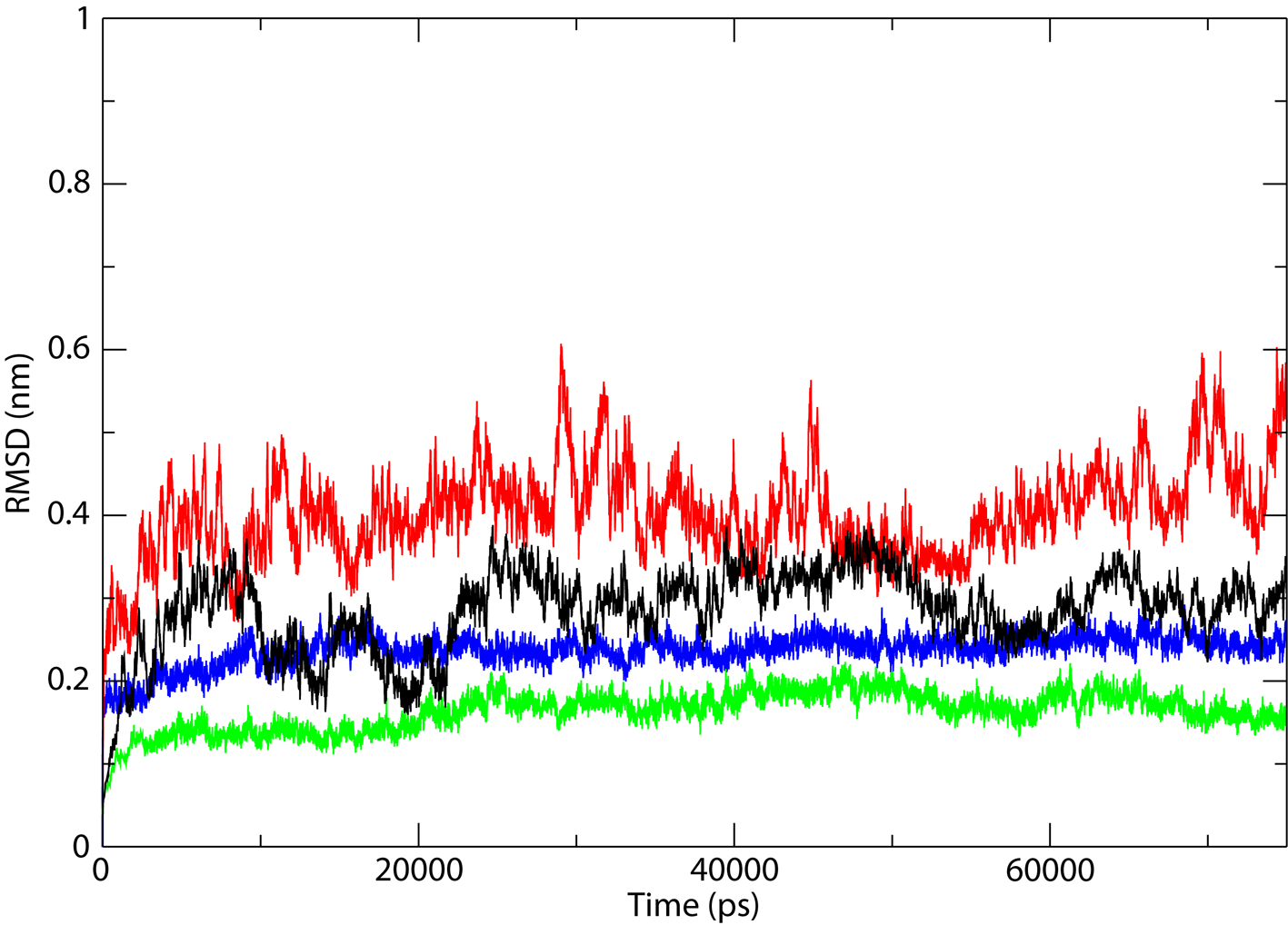

Supplement: Figure S3 — Cα atoms Root Mean Square Deviation (RMSD) calculated as a function of time for the hTop1 with (black line) and without the linker domain (green line), for the hTop1(pf-Linker) with (red line) and without the linker domain (blue line). (TIF) [file pone.0068404.s003.tif]

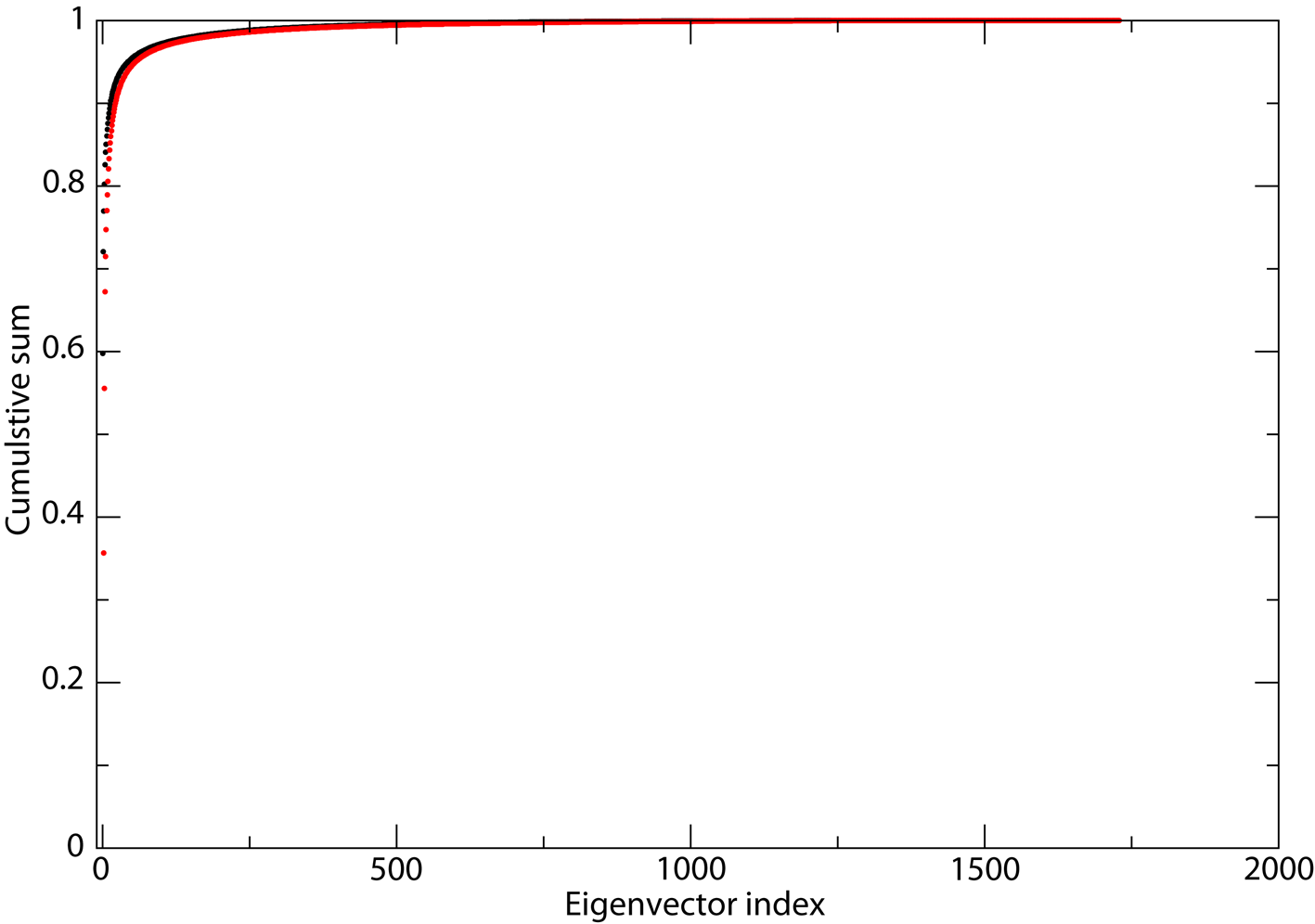

Supplement: Figure S4 — Cumulative percentage of motion as a function of eigenvectors for the Cα atoms of the hTop1 (black line) and hTop1(pf-Linker) complex (red line). (TIF) [file pone.0068404.s004.tif]

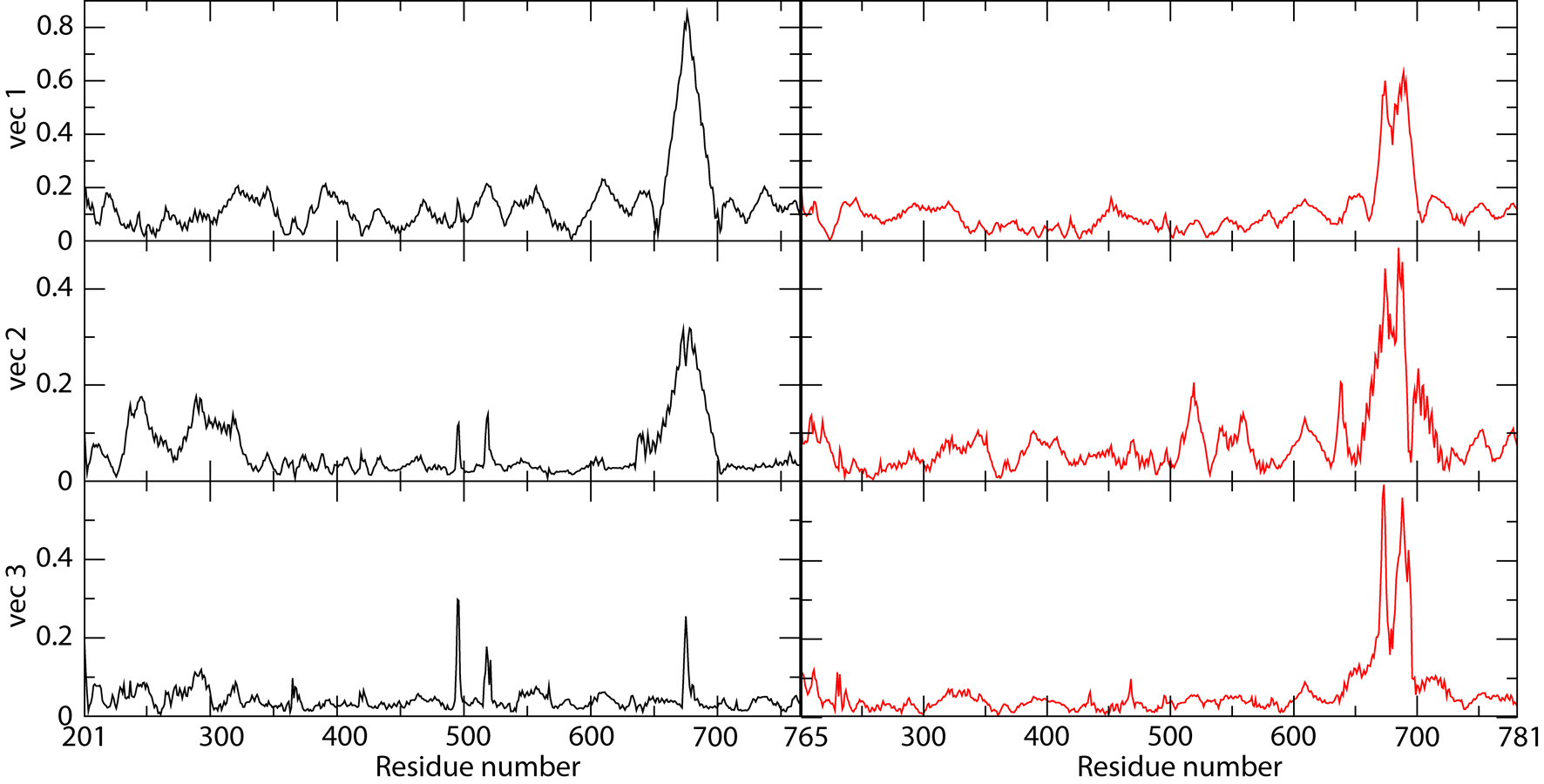

Supplement: Figure S5 — Root Mean Square Fluctuations along the first three eigenvectors for the hTop1 (left panel, black line) and hTop1(pf-Linker) (right panel, red line) enzyme. (TIF) [file pone.0068404.s005.tif]
